# Bryophyte-Cyanobacteria Associations during Primary Succession in Recently Deglaciated Areas of Tierra del Fuego (Chile)

**DOI:** 10.1371/journal.pone.0096081

**Published:** 2014-05-12

**Authors:** María Arróniz-Crespo, Sergio Pérez-Ortega, Asunción De los Ríos, T. G. Allan Green, Raúl Ochoa-Hueso, Miguel Ángel Casermeiro, María Teresa de la Cruz, Ana Pintado, David Palacios, Ricardo Rozzi, Niklas Tysklind, Leopoldo G. Sancho

**Affiliations:** 1 Dept. de Biología Vegetal II, Universidad Complutense de Madrid, Madrid, Spain; 2 School of Environment Natural Resources and Geography, Bangor University, Bangor, Wales, United Kingdom; 3 Dept. de Biología Ambiental, Museo Nacional de Ciencias Naturales, MNCN-CSIC, Madrid, Spain; 4 Biological Sciences, Waikato University, Hamilton, New Zealand; 5 Dept. de Biogeografía y Cambio Global, Museo Nacional de Ciencias Naturales, Madrid, Spain; 6 Departamento Edafología, Universidad Complutense de Madrid, Madrid, Spain; 7 Departamento Geografía Física, Universidad Complutense de Madrid, Madrid, Spain; 8 Sub-Antarctic Biocultural Conservation Program, University of North Texas, Denton, Texas, United States of America; 9 Institute of Ecology and Biodiversity, Universidad de Magallanes, Puerto Williams, Chile; 10 School of Biological Sciences, Bangor University, Bangor, Wales, United Kingdom; Lakehead University, Canada

## Abstract

Bryophyte establishment represents a positive feedback process that enhances soil development in newly exposed terrain. Further, biological nitrogen (N) fixation by cyanobacteria in association with mosses can be an important supply of N to terrestrial ecosystems, however the role of these associations during post-glacial primary succession is not yet fully understood. Here, we analyzed chronosequences in front of two receding glaciers with contrasting climatic conditions (wetter *vs* drier) at Cordillera Darwin (Tierra del Fuego) and found that most mosses had the capacity to support an epiphytic flora of cyanobacteria and exhibited high rates of N_2_ fixation. Pioneer moss-cyanobacteria associations showed the highest N_2_ fixation rates (4.60 and 4.96 µg N g^−1^ bryo. d^−1^) very early after glacier retreat (4 and 7 years) which may help accelerate soil development under wetter conditions. In drier climate, N_2_ fixation on bryophyte-cyanobacteria associations was also high (0.94 and 1.42 µg N g^−1^ bryo. d^−1^) but peaked at intermediate-aged sites (26 and 66 years). N_2_ fixation capacity on bryophytes was primarily driven by epiphytic cyanobacteria abundance rather than community composition. Most liverworts showed low colonization and N_2_ fixation rates, and mosses did not exhibit consistent differences across life forms and habitat (saxicolous *vs* terricolous). We also found a clear relationship between cyanobacteria genera and the stages of ecological succession, but no relationship was found with host species identity. Glacier forelands in Tierra del Fuego show fast rates of soil transformation which imply large quantities of N inputs. Our results highlight the potential contribution of bryophyte-cyanobacteria associations to N accumulation during post-glacial primary succession and further describe the factors that drive N_2_-fixation rates in post-glacial areas with very low N deposition.

## Introduction

Cryptogamic covers account for nearly half of the biological nitrogen (N) fixation on land [1], representing an important supply of N to terrestrial ecosystems. In particular, dinitrogen (N_2_) fixation by cyanobacteria-moss associations has been found to contribute significantly to the N economy in high-latitude ecosystems [2]–[6] where it can represent the main source of *new* N [7]–[10]. For example, moss-associated cyanobacteria can provide 2–58% of the N input to Arctic ecosystems and in Antarctica the percentage can reach up to 84% [11]. In boreal forests, up to 60% of N can come from N_2_-fixation in mosses [12]. In early primary succession, however, the role of cyanobacteria-moss associations on N inputs has received considerably less attention, despite bryophytes being often the first plants to colonize newly exposed terrain [13]. The lack of studies is particularly marked in temperate and subpolar regions of the Southern Hemisphere, where bryophytes account for more than 50% of the plant species [14].

Rates of N_2_ fixation in moss associated with cyanobacteria can be highly variable. Previous studies in the 1980s and early 1990s have shown that N_2_ fixation varies greatly among bryophyte species [8], [15]–[17] which may be due to differences in the microhabitat characteristics where the mosses grow [2]. Moderate increases in temperature leads to a general increase in N_2_ fixation [18] whereas shading [19] and N fertilization (e.g. atmospheric N deposition or throughfall) results in reduced rates of N_2_ fixation in these associations [4], [12]. Moisture is also considered a major environmental factor influencing N_2_-fixation on moss-cyanobacteria associations, with a positive relationship between water availability and N-fixation rates [3], [20]. The diversity and density of cyanobacteria species associated with mosses have also been described as important factors explaining the variability in N_2_ fixation rates [21], [22]. However, little is still known about the existence of species-specific association between bryophyte and cyanobacteria species [23], although some studies have shown that host species identity could be a stronger driver of epiphytic cyanobacteria diversity and composition [22]. While these studies have already started to elucidate the factors that drive N_2_-fixation rates, the influence of bryophyte-cyanobacteria associations on the biogeochemical cycling of N and, in particular, during early primary succession still needs further assessment.

In Tierra del Fuego, glacial retreat has been occurring since the end of the Little Ice Age (around 1750 to 1850) [24], exposing new terrain for ecosystem development by the process of primary succession. Glacier forelands are typically nutrient-poor [25]. However, our preliminary studies at the Cordillera Darwin (Tierra del Fuego) found rapid rates of vegetation growth and quick succession to stages dominated by *Nothofagus* tree species [26]. These tree-dominated stages were present on the southern side of the mountain range after 34 years of soil surface exposure [26], compared to 80 years on the northern side of the mountain range (Palacios *et al*., unpublished). As inputs of N through atmospheric deposition are very low in such high latitude region, possibly <0.7 kg N ha^−1^ yr^−1^ [27], biological fixation can greatly exceed bulk precipitation. For example, biological N fixation inputs from bryophytes during early succession, e.g. [28] reached 22 kg N ha^−1^ yr^−1^ in a New Zealand glacier foreland. Therefore, we considered that N_2_ fixation and its interaction with other biotic and abiotic factors could be a key driver of early plant succession in recently deglaciated areas of Tierra del Fuego. The combination of three attributes: 1) the difference in the speed of ecological succession found between the two glaciers on southern and northern slopes; 2) the strong climatic gradients characteristic of this area: with high rates of precipitation on the southwestern side and dry conditions on the northern side of the mountain range [29]; and 3) the low rates of anthropogenic N deposition, make the Cordillera Darwin an unique site to further elucidate the potential drivers of N_2_ fixation rates on bryophyte-cyanobacteria associations and its impact on C and N accumulation during post-glacial primary succession.

Specifically, we aimed to evaluate the role of bryophyte-cyanobacteria associations during post-glacial primary succession because (i) moss establishment is a positive feedback process that enhance N inputs in newly exposed terrain [13] and (ii) moss-associated cyanobacteria can be an important contributor to N inputs through the process of N_2_ fixation [28], [30]. Our aims were (1) to describe how soil properties and N_2_ fixation related parameters change along two pro-glacial chronosequences at the Cordillera Darwin (Tierra del Fuego), (2) how species identity (both bryophytes and cyanobacteria), climatic conditions (wetter *sv*. drier), and the stage of ecological succession, influence N_2_ fixation rates of bryophyte-cyanobacteria associations, and thereby ecosystem N accumulation, and (3) whether any of these associations are species specific.

## Materials and Methods

### Ethics Statement

The Chilean National Forestry Corporation (CONAF) issued the scientific collecting permit for each location considered in this study. This study did not involve endangered or protected species.

### Site description and chronosequence establishment

**Figure 1:**
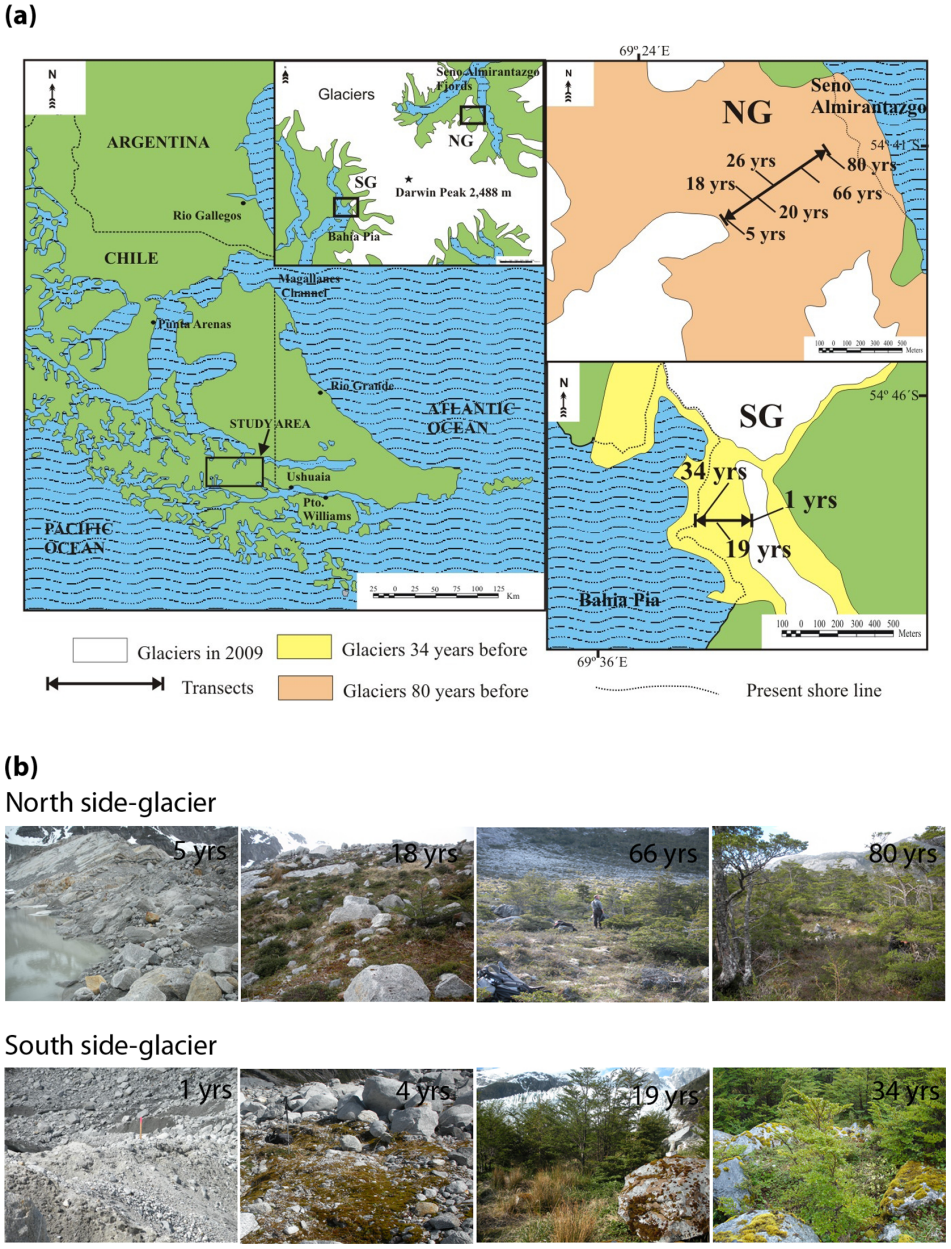
Study area and chronosequence description. (a) Map of the study area at the Cordillera Darwin in the southwestern part of Tierra del Fuego (Chile) showing the location of the south-side (SG) and north-side (NG) glaciers on each side of the mountain range. (b) Four representative sites along north-side glacier and south-side glacier chronosequences at Cordillera Darwin. At both glaciers, the younger site corresponds to bare ground close to the glacier front whereas the older site was covered within *Nothofagus* forests. Years since deglaciation per site are included in the picture.

The study area is located at the Cordillera Darwin in the southwestern part of Tierra del Fuego (Chile) (Fig. 1a). Around 80% of the mountain range is glaciated. One glacier was selected on each side of the mountain range for the present study; both glaciers have been receding steadily for several decades and present a well-defined sequence of moraine bands. The south-side glacier, SG (Pia Glacier, 54°46′S 69°40′W) flows into the east arm of Bahia Pia; plant succession goes from bare ground to full forest (stage of succession dominated by *Nothofagus* spp. >10 m high) in 34 years [26]. At the north-side glacier, NG (Parry Glacier, 54°41′S 69°23′W) the succession is slower and the equivalent stage of succession dominated by *Nothofagus* appears after 80 years of soil exposure (Palacios *et al*., unpublished data). A characteristic feature of the area is the steep meteorological gradients across the Cordillera Darwin with heavy precipitation and solid cloud cover being typical over the southern and western fjords, whilst to the north and east conditions are much drier [29]. This results in a stark contrast in annual precipitation between both glaciers. Precipitation rates where SG flows, Bahia Pia (1600 mm) are twice as high as at where NG flows, Seno Almirantazgo fjord (800 mm) [29]. Mean annual air temperature, however, is relatively constant over the entire region, with only a slight drop from 5.9°C at Punta Arenas to 4.5°C at Bahia Pia [31].

The chronosequence in each glacier has been established by using several independent techniques to date surfaces: aerial photographs, dendrochronology, (sampling tree rings), and lichenometry (dating of rock surface from lichens growth) utilizing the growth rate and thallus size of the lichen *Rhizocarpon geographicum* (L.) DC [26].

Forest vegetation in the area consists of *Nothofagus antarctica* (G. Forst.) Oerst., and *N. betuloides* (Mirb.) as dominant tree species. After glacier retreat, the successional sequence at NG is characterized by pioneer lichens (*Placopsis perrugosa* (Nyl.) Nyl., *Rhizocarpon geographicum* (L.) DC., *Xanthoria candelaria* (L.) Th. Fr.) and mosses (*Dendroligotrichum squamosum* (Hook. f. & Wilson) Cardot and *Andreaea* spp.), followed by dwarf shrubs (*Empetrum rubrum* Wahl. Ex Willd., *Gaultheria mucronata* (L. f.) Hook. & Arn.), perennial herbs (*Gunnera magellanica* Lam) and moss (*Racomitrium* spp.) vegetation assemblage. At SG the successional sequence goes from pioneer lichen (same species as in NG plus *Placopsis stenophylla* (Hue) I.M. Lamb and *Placopsis pycnotheca* I.M. Lamb) and the moss *Ditrichum cylindricarpum* (Müll. Hal.) F. Muell, followed by perennial herbs vegetation assemblage (*Gunnera magellanica* Lam and *Uncinia tenuis* Poepp. ex Kunth) and the development of *Nothofagus* spp. forest (Fig. 1b).

### Sample collection and transport

Field work took place in 2009 during the summer season which corresponds to the active period for N_2_ fixation at high southern latitudes [32]. Sampling sites were established at increasing surface ages in front of both glaciers. There were six sites at SG with surface ages of 1, 4, 7, 10, 19 and 34 yrs and six sites at NG with surface ages of 5, 18, 20, 26, 66 and 80 yrs. At both glaciers, the younger site corresponds to bare ground close to the glacier front whereas the older site was covered within *Nothofagus* forests. Three transects were randomly established along the chronosequence in each glacier foreland and one independent sample was collected in each transect per site (surface age) so that there were three replicates per surface age for soil, each bryophyte species and cyanobacteria measurements.

#### Soil sampling

200 g of soil samples were collected using the core method, samples were collected from the upper 0–4 cm of the soil. Samples were placed into, and sealed in, suitably sized plastic bags. Collected samples were immediately frozen at −20°C and returned frozen to the Universidad Complutense de Madrid for soil characterization.

#### Bryophyte sampling

The dominant bryophyte species at each site (surface age) along the chronosequence were collected. In total 16 bryophyte species (including both mosses and liverworts) with varying morphologies, life forms and habitats (saxicolous and terricolous species) were selected. These included 10 mosses *Dendroligotrichum squamosum* (Hook. f. & Wilson) Cardot, *Andreaea laxifolia* (Hook. f. & Wilson), *A. alpina* (Hedw.), *Acroschisma wilsonii* (Hook. f.) A. Jaeger, *Racomitrium didymum* (Mont.) Lorentz, *R. laevigatum* (A. Jaeger), *R. lanuginosum* (Schimp. ex R.S. Williams), *R. subcrispipilum* (Müll.Hal.) A. Jaeger, *Dicranoloma chilense* (De Not.) Ochyra & Matteri and *Ditrichum cylindricarpum* (Müll. Hal.) F. Muell; and 6 leafy liverworts *Cryptochila grandiflora* (Lindenb. & Gott.) Grolle, *Heteroscyphus magellanicus* (Steph.) Engel & Schust., *Blepharidophyllum densifolium* (Hook.) Ångström ex C. Massal., *Anastrophyllum involutifolium*, *Clasmatocolea humilis* (Hook. f. & Tayl.) Grolle and *Chiloscyphus leptanthus* (Hook. f. & Tayl.) Engel & Schust. Eight species were sampled at more than one site and the other eight were sampled at a single site on the chronosequence. A full description of bryophyte species sampled by site are shown in (Table S1). Monospecific patches of fresh bryophytes species were collected, air dried and sent to the Universidad Complutense de Madrid in paper bags for analysis that included: (1) rates of N_2_ fixation, (2) quantification of the abundance of epiphytic cyanobacteria, and (3) abundance of stable isotopes (^14^N and ^15^N) and shoot N and C contents.

#### Cyanobacteria sampling

The diversity and distribution of cyanobacteria associated with bryophytes at each site (surface age) was determined by molecular analysis. A second set of bryophyte samples (around 10 shoots per species) were collected in the same bryophyte patches described above. Collectors used disposable laboratory latex gloves when taking samples. Samples were placed into, and sealed in suitably sized plastic bags. Collected samples were immediately frozen at −20°C and returned frozen to the Museo Nacional de Ciencias Naturales in Madrid (CSIC) where DNA from epiphytic cyanobacteria was extracted and analysed.

### Soil chemical properties

Samples were stored at −20°C. Soils were air-dried, sieved (<2 mm) homogenized before conducting the following analysis:

#### Soil pH

Soil pH was measured in water suspension (soil∶water, 1∶2.5) [33].

**Total soil N (TN) and total soil carbon (TC)** were analysed in an autoanalyser system LECO CNS 2000I.

#### Soil extractable nitrogen

Soil nitrate (NO_3_-N) and nitrite (NO_2_-N) were extracted by shaking soil aliquots (30 g DW) in (1∶5) water [33], filtered and analysed by ionic chromatography Metrohm 761 compact IC. Ammonium (NH_4_-N) was extracted by shaking 10 g of soil in 50 ml of 0.5 M K_2_SO_4_ for 30 min, filtered and colorimetrically assessed [34].

### Nitrogen-fixation measurements

Prior to N_2_-fixation measurements, all bryophyte samples were reactivated for 48 h in a growth chamber, photosynthetic photon flux density (PPFD) of 100 µmol m^−2^ s^−1^ over the waveband 400–700 nm, 10°C, with a 18 ∶ 6 h phototoperiod (light ∶ dark) and maintained hydrated to ensure recovery (confirmed by chlorophyll fluorescence, data not shown). N_2_-fixation rates of bryophyte-cyanobacteria associations were estimated under controlled conditions using the acetylene reduction assay (ARA) [35]. Revitalized shoot apices (around 10–15 shoots or 0.07–0.20 dry weight basis) were placed into 10 ml glass incubation vessels and sealed with a rubber septum. 1 ml of headspace was removed and replaced by 1 ml of acetylene (generated from CaC_2_ and water), resulting in headspace enrichments of 10–20% (enrichment was determined individually for each species allowing for the different vial headspaces, see in (Appendix S1). Four samples were randomly chosen as control samples and were treated in the same manner except for the headspace substitution; another set of four samples with deionized water and acetylene was used as blank samples to assess the concentration of ethylene in the acetylene used. All incubation vessels were placed in a growth chamber (200 µmol m^−2^ s^−1^ PAR, 10°C) and incubation took 18 h. Before incubation, acetylene reduction was demonstrated to be linear over an 18-h period in 10 samples with known different cyanobacteria abundance (data not shown). Ethylene resulting from the reducing activity was measured by gas chromatography. N_2_-fixation rates were calculated as nanomoles of acetylene reduced per gram bryophyte dry weight per hour (nmol C_2_H_2_ g^−1^ dw h^−1^). Theoretical stoichiometric ratio of acetylene to nitrogen (3∶1) is recommended to be corrected by parallel uptake of labelled ^15^N_2_ for each organism and also, possibly, for location [36], [37]. Thus, to estimate N_2_ fixation rates (^15^N_2_ uptake), consecutive incubations of the same samples with ^15^N_2_ after ARA was performed following [9]. 2 ml of headspace were replaced by 2 ml ^15^N_2_ gas (98 atom % ^15^N enriched), resulting in headspace enrichments of 20–30% and, after 18 h incubation at the same conditions as ARA, samples, were immediately oven dried (60°C for 48 h), ground in a ball mill and sent for ^15^N enrichment and total N analysis (UCDavis Stable Isotope Facility, University of California, Davis, USA). Three control samples for each bryophyte-cyanobacteria association were used to determine the natural abundance ^15^N. The amount of N fixed was calculated according to [6], [38]. N_2_-fixation rates were calculated as micrograms of ^15^N incorporated per hour per gram of bryophyte on a dry weight basis and expressed as micrograms of fixed N per day (µg N g^−1^ dw bryophyte d^−1^).

ARA was highly correlated with ^15^N_2_ uptake (*N* = 89, *R^2^* = 0.689 *P*<0.001). ARA measurements were used to compare N_2_ fixation rates between taxa and rates reported by other authors. ^15^N_2_ uptake was used to evaluate the potential impact of N_2_ fixation on ecosystem nitrogen (N) accumulation.

### Stable isotope analysis and shoot N and C content

All isotopic analyses were conducted at the UCDavis Stable Isotope Facility, Department of Plant Sciences, University of California, Davis, USA. *δ*15N analyses were performed by elemental analyser/continuous flow isotope ratio mass spectrometry using a ANCA-GSL elemental analyser interfaced coupled with a PDZ Europa 20-20 isotope ratio mass spectrometer. Atom% was calculated from *δ* ^15^N values. Total tissue C and N was also analysed.

### Cyanobacteria abundance, composition and phylogenetic analysis

#### Cyanobacteria abundance by HPLC analysis of echinenone

Bryophyte samples (∼3–10 cm^2^ of fresh tissue per sample depending on the species) were deep-frozen until pigment analyses. Pigments were extracted in 95% cool acetone in the presence of sodium ascorbate. Echinenone (a cyanobacterial pigment used as a proxy of cyanobacteria abundance) was separated by HPLC according to the method of [39] slightly modified. After passing through a 0.45 µm nylon filter, 25 µL of the extract was injected into a C18 column. The mobile phase rate was 1.2 mL min^−1^ and the elution time lasted 30 min. Solvents for HPLC analysis were degassed before use by bubbling helium. The HPLC system was equipped with a photodiode array detector. For peak identification and quantification, a pure commercial standard was used. The relative abundance of cyanobacteria was expressed as the amount of echinenone on a bryophyte surface area basis (nmol echinenone/cm^2^ bryophyte). Digital pictures of each sample were taken before freezing and the surface area of each sample was calculated using a image analysis program.

#### DNA extraction and polymerase chain reaction (PCR), denaturing gradient gel electrophoresis (DGGE)

For cyanobacteria DNA extraction, bryophyte samples (around 200 mg dry weight) were introduced into 15 ml falcon tubes with distilled water and vigorously shaken (maximum speed) for 10 minutes in a vortex using a falcon tube adapter to release the epiphytic cyanobacteria from bryophytes. The extracted cyanobacteria contained in the water were removed with a 5 ml pipette and cells were concentrated by centrifugation in an ultracentrifuge at 3000 rpm for 5 min. The concentrated cyanobacteria cells were used for DNA extraction.

Denaturing gradient gel electrophoresis (DGGE) of cyanobacterial PCR-amplified 16S rRNA gene fragments was used to survey the genetic diversity of isolated epiphytic cyanobacteria. Total genomic DNA for DGGE analysis was extracted in three replicates from the cyanobacteria isolates samples using CTAB extraction method described in [40]. A fragment of the cyanobacterial 16S rRNA gene suitable for DGGE analysis was amplified in each sample from total genomic DNA using the following primer pair: CYA359fGC and CY781r [41]. Each 25 µl volume of PCR mix [75 mM Tris pH 9.0/50 mM KCl/20 mM (NH_4_)_2_SO_4_] contained 1 unit of Taq polymerase, 0.2 mM of each of the four dNTPs, 0.4 mM of each primer, 100 mg of bovine serum albumin, 1.5 mM of MgCl_2_, 5 ml de 5xTaq Master PCR enhancer (Prime) and ca 10–50 ng genomic DNA. Annealing conditions were 60°C. The DGGE system was used for the analysis of cyanobacterial fragments of 16S rRNA gene according to the manufacturer's instructions). Acrylamide gels (6%) with a 30 to 60% urea-formamide denaturing gradient were prepared. Lanes were loaded with 22 µl of PCR product, run at a constant 200 V for 7 h at 60°C, and EtBr stained to visualize and photograph the resultant bands according to [42]. The most predominant bands were excised and incubated 1 h at 60°C in water before PCR amplification. The eluted DNA was reamplified as described above but using primers devoiding the GC clamp. The PCR products were cleaned on Quick Spin columns. Both complementary strands were sequenced separately at the MACROGEN sequencing company.

**Figure 2:**
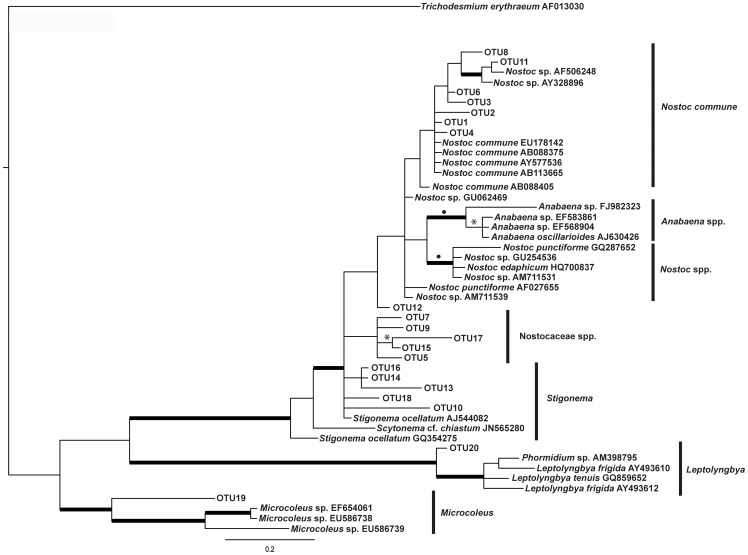
Maximum-likelihood phylogenetic tree. Maximum-likelihood phylogenetic tree (50% majority rule consensus tree) from alignments of 16S rRNA genes showing the phylogenetic relationships among all OTUs of epiphytic cyanobacteria found on bryophytes collected along the chronosequence in both glacier and sequences with known taxonomy, for Accession numbers (Table S1).

For sequence analysis, we obtained closely related sequences with known taxonomy using Blast searches in Gen Bank. Later, sequences obtained from DGGE bands were reduced to OTUs in *mothur* v.1.28 [43] using a cutoff of 0.01% [44]. A reference sequence for each OTU was chosen for subsequent analysis by means of the command *get.outrep* in *mothur* v.1.28. 20 sequences corresponding to each of the 0.01% cut-off OTUs were aligned with a total of 29 sequences retrieved from the GenBank (Table S2). Alignment of all sequences were carried out in Muscle 3.8.31 [45] and visually checked in Bioedit 7.0.5.2 [46]. Nucleotide substitution model was statistically selected with the help of jModelTest [47], program available at http://darwin.uvigo.es). Model selection was made according to the Akaike′s information criterion (AIC, [48]; the General Time Reversible substitution model [49], with estimation assuming a gamma distribution (GTR+G) had the lowest –lnL value. Phylogenetic relationships among OTUs and sequences with known taxonomy were studied by means of Maximum Likelihood (ML) and Bayesian phylogenetic inference. ML analysis were carried out in PHYML 3.0 [50] through the webserver http://www.atgc-montpellier.fr/phyml/ using 1,000 bootstrap repetitions in order to assess node support. Bayesian analyses were carried out in Mr. Bayes 3.1.2 [51] running eight different chains and 5,000K generations. 50% majority rule consensus tree is shown in (Fig. 2).

Sequences generated in this study were deposited in GenBank under accession numbers KJ576732-KJ576783.

### Statistical and data analyses

We calculated the Shannon index (*H′* = -Σ (*ni/N*) ln (*ni/N*) [52], where *ni* is the number of times we found each *i*th OTU and *N* is the total number of unique OTUs (*N* = 20) found in the study area, in order to evaluate changes in diversity of epiphytic cyanobacteria along the chronosequence (*H′_site_*) and between bryophytes species (*H′_species_*).

To be able to compare parameters between glacier chronosequences with different sampling site ages we first tested if the different stages of ecological succession, identified by the soil development transformation trends observed along the chronosequence, were comparable between the two glacier chronosequences; we used TC, TN and soil pH as descriptors of soil development status at each site and extracted one general factor component of soil development using principal component analysis [53]. The resulting scores were tested with two-way ANOVA to detect differences on the stage of ecological succession along the chronosequence and between glaciers, with sampling sites (0 to 5) and glacier location (north *sv*. south) as fixed factors. These analyses indicated that the two chronosequences were comparable despite the different absolute soil ages where the samples were collected (Fig. S1). Differences between glaciers on soil chemical properties, N_2_-fixation rates (^15^N_2_ uptake), abundance of epiphytic cyanobacteria, and C : N ratio in bryophyte tissue were then tested with two-way ANOVAs. All data were tested for homoscedasticity (Levene's test for equal variances) and normality (IBM SPSS statistics, Armonk NY, USA). When data did not meet these assumptions, they were rank or Log_10_ +1 transformed prior to statistical analyses. Analyses were carried out with IBM SPSS Statistics 19 (IBM SPSS statistics, Armonk NY, USA).

A fundamental assumption about chronosequences is that the communities and ecosystems of the younger sites are developing in the same temporal sequence as the older sites developed (termed a space-for-time substitution). At any location on the chronosequence the vegetation present represents what will eventually form on younger sites and which will, itself, change to that found on the older sites present. As such, the assumption of independence is violated. We used non-parametric Krukall-Wallis test to evaluate changes of parameters measured at the different stages of ecological succession (mean values per site) along each glacier chronosequence separately, using stage of ecological succession (sampling site) as the grouping factor. When significant differences were found, Mann–Whitney U-test post hoc tests were run to detect differences between sites. Bonferroni's correction was used to determine the level of significance of the post hoc tests.

At species level, we used conditional inference trees (CTREE) [54] to assess and visualise the relative importance of bryophyte species identity (species effect), climatic conditions (glacier location: north *sv*. south), and stage of ecological succession (sampling site effect) in determining N_2_ fixation capacity and cyanobacteria abundance on bryophytes. CTREE work by first testing the null hypothesis of independence between the predictors (here bryophyte species, glacier and site) and response variable (here N_2_ fixation and cyanobacteria abundance); if dependence is found, then determining the best split value for the predictor with the strongest effect on the response; and third, repeating the first two steps with each branch until independence cannot be rejected. Relationships are represented as dichotomous trees with nodes indicating split points for significant predictor variables, and terminal nodes for the final groups of responses. Being nonparametric, CTREE make no assumptions about the data, and are appropriate where nonlinear relationships between predictive and response variables and complex interactions between variables, such as covariance, may be expected [54]. CTREE were constructed in R 3.0.1 (R Development Core Team, 2013) with the package PARTY [55], with 10,000 resamples of Bonferroni-adjusted α = 0.05 significance levels and with at least one observation into each daughter node. N_2_ fixation and cyanobacteria abundance data were log +1 transformed and site included as an ordered categorical variable.

Linear regression analysis was used to evaluate whether abundance of epiphytic cyanobacteria or its diversity on host bryophyte (*H′_species_*) had a larger influence on the rate of N_2_ fixation.

Correspondence analysis in each glacier separately were used to graphically examine the relationships between OTUs of cyanobacteria and the different stages of ecological succession and also to detect selectivity associations between cyanobacteria OTUs and bryophyte species (6 species with cyanobacteria colonies sampled at more than one site were used in this analyses). Analysis were carried out in R 3.0.1 (R Development Core Team, 2013) using the package *ca* [56].

## Results

### Genetic diversity of isolated epiphytic cyanobacteria

A total of 52 bands (18 from NG and 34 from SG) were recovered from DGGE analyses and subsequently sequenced, producing 28 unique sequences. After their collapse into OTUs (0.01% cutoff), 20 cyanobacteria putative taxa were obtained. The phylogenetic analysis of the relationships between these OTUs and sequences with known taxonomy obtained from the GenBank is depicted in Fig. 2. Seven 0.01% cutoff OTUs could be assigned to the species aggregate *Nostoc commune*. Six OTUs (5, 7, 9, 12, 15, 17) were assigned to unidentified Nostocaeae because no clear relationship was found in the phylogenetic tree with any certain genus. A similar case is presented for OTUs 10, 13, 14, 16, and 18, which did not show a clear and well supported clade with sequences of *Stigonema* included in the phylogenetic analysis. Nevertheless, BLAST searches of these OTUs recovered sequences identified as *Stigonema*. In both cases, the most likely explanation for such lack of phylogenetic support in our tree for these two OTU groups is the shortness of fragment of the 16S gene used in the analyses. Finally, OTU 20 is clearly and statistically supported related to *Leptolymbya* and OTU 19 is likewise related to *Microcoleus* (Fig. 2).

### Soil properties of glacier forelands

**Figure 3:**
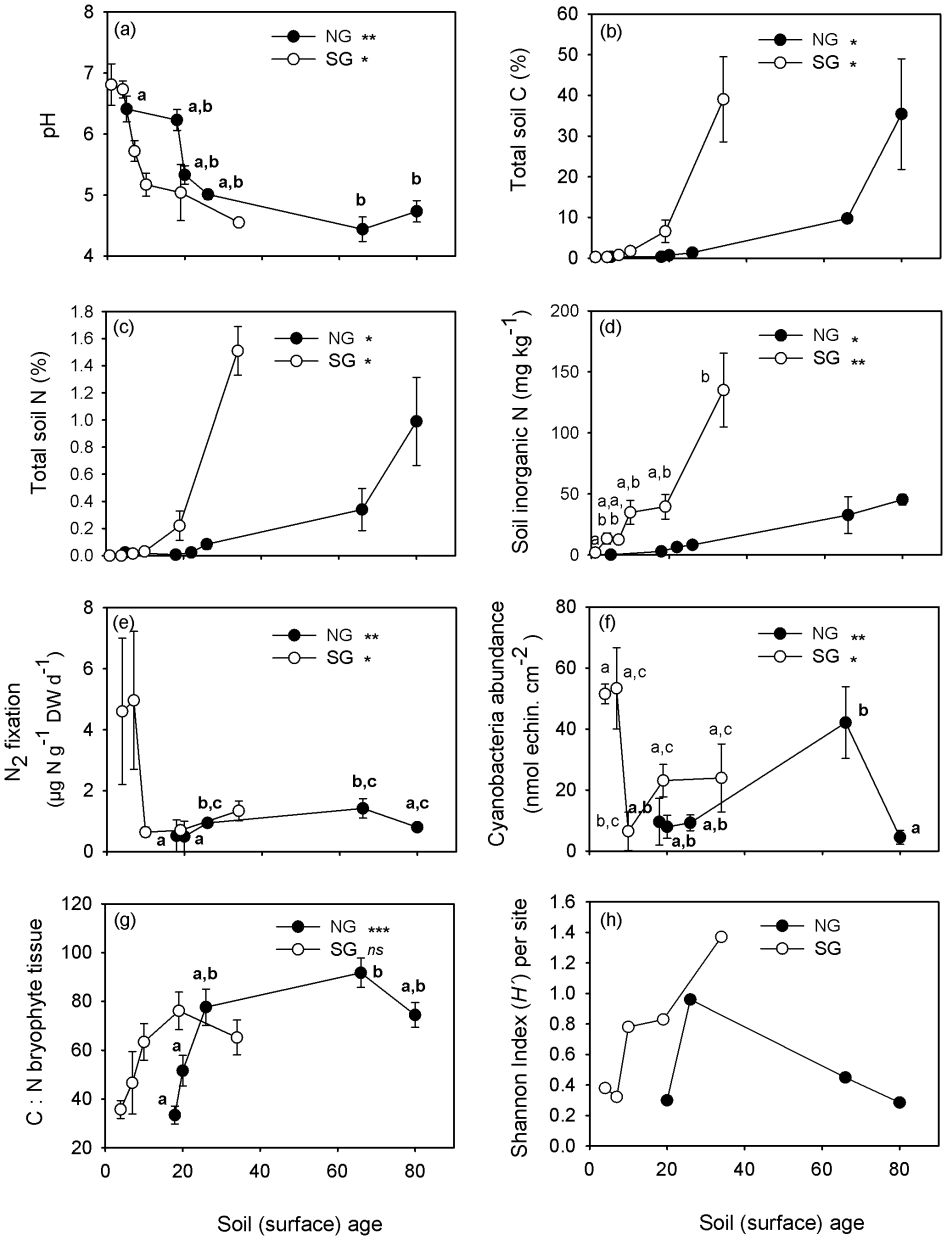
Patterns of soil properties and N_2_ fixation related parameters along chronosequences. Site characteristics along the chronosequence in the north-side glacier (NG: black circles) with site soil (surface) age of 5, 18, 20, 26, 66 and 80 yrs and south-side glacier (SG: white circles) with site soil (surface) age of 1, 4, 7, 10, 19 and 34 yrs. At both glaciers, the younger site corresponds to bare ground close to the glacier front whereas the older site was covered within *Nothofagus* forests. Significant changes of parameters along each glacier chronosequence is indicated by *, *p*<0.05*, *p*<0.01**, *p*<0.001*** (Krukall-Wallis test). Different letters indicate significant differences between site ages after Mann–Whitney U-test post-hoc (Bonferroni corrected), bold letters are used for NG. Statistical differences on soil properties and N_2_ fixation related parameters between the two glacier chronosequences are indicated in the result section.

Overall comparisons between both glaciers (two-way ANOVAs) showed no differences on pH, TC and TN between glaciers (NG *vs* SG) and higher concentration of inorganic N at SG (ANOVA *P* = 0.037). At NG, the concentration of inorganic N in young soils (younger than 26 years) was lower (from 0.16 to 8.23 mg kg^−1^) compared to concentrations observed in soils at SG very early after glacier retreated (13.42 mg kg^−1^ after 4 years soil exposure and 34.83 mg kg^−1^ after 10 years, Fig. 3d). Within the *Nothofagus* forests inorganic N concentration in the soil was nearly 3-fold higher at SG (135.08 mg kg^−1^) compared to NG (45.28 mg kg^−1^). All soil parameters showed a significant overall site effect (ANOVA *P*<0.001) and no interaction between glacier location (north *sv*. south) and site (0 to 5). Within each chronosequence (non-parametric tests), soil pH decreases from 6.4 to 4.4 in NG (*P*<0.01) and from 6.81 to 4.55 in SG (*P*<0.05), with the higher values always found in the younger soils (Fig. 3a). TC increased from very low values (0.33% at NG and 0.24% at SG) to over 35% at NG (*P*<0.05) and 39% at SG (*P*<0.05) in the oldest soils (80 and 34 years of soil exposure respectively) inside the *Nothofagus* spp. forest (*Nothofagus*-dominated state, Fig. 3b); TN also increased along the chronosequence from 0.02% in NG and undetectable levels in SG to 0.99% in NG (*P*<0.05) and 1.51% in SG (*P*<0.05) inside the forest site (Fig. 3c). Soil extractable inorganic N (NO_3_^−^+NO_2_^−^+NH_4_^+^) followed a similar trend increasing with time since deglaciation at both glaciers (*P*<0.05 for NG and *P*<0.01 for SG, Fig. 3d).

### Nitrogen-fixation, cyanobacteria abundance and shoot C : N contents

N_2_ fixation rates (^15^N_2_ uptake) and epiphytic cyanobacteria relative abundance, when averaged across all bryophyte species per site, were higher at SG compare to NG (ANOVA *P*<0.001 and *P*<0.01 respectively). A significant overall interaction (ANOVA *P*<0.001) between glacier locations (north *sv*. south) and the stage of ecological succession (sampling site:1 to 5) suggested that variation of N_2_ fixation rates and cyanobacteria abundance along each chronosequence differed between glaciers. At NG, N_2_ fixation rate (Fig. 3e) showed an optimum increasing from the lowest rates at 18 and 20 year old sites (0.52 and 0.5 µg N g^−1^ DW bryo. d^−1^, respectively) to rates that were 2-fold higher in intermediate-aged sites (0.94 and 1.42 µg N g^−1^ DW bryo. d^−1^, 26 and 66 years old site respectively) and then declined in the 80 years old forest site (0.80 µg N g^−1^ DW bryo. d^−1^). Epiphytic cyanobacteria abundance (Fig. 3f) followed a similar trend with the highest concentration at 66 years old site (42.09 nmol echin. cm^−2^) and lowest concentration at 80 and 20 years old soil (4.5 and 7.94 nmol echin. cm^−2^). In contrast, at SG the highest N_2_ fixation rate and cyanobacteria abundance was found in young soils (4.60 and 4.96 µg N g^−1^ DW bryo. d^−1^ and 51.50 and 53.33 nmol echin. cm^−2^, at 4 and 7 year old sites respectively) compared to older sites (ranging from 0.64 to 1.34 µg N g^−1^ DW bryo. d^−1^ and 6.46 to 23.96 nmol echin. cm^−2^, at soil age 10 to 34 years old; Fig. 3e,f).

Shoot C : N ratios of bryophyte tissue, when averaged across all bryophyte species per site, did not differed between glacier (ANOVA *P* = 0.165) but change with soil age (ANOVA *P*<0.001). Shoot C : N increased with time since deglaciation at NG (*P*<0.001) with the lower values always found in the younger soil (Fig. 3g). At SG shoot C : N ratios showed the same pattern but the effect of soil age was not significant (Fig. 3g).

### Differences on bryophyte N_2_ fixation capacity and cyanobacteria abundance

**Figure 4:**
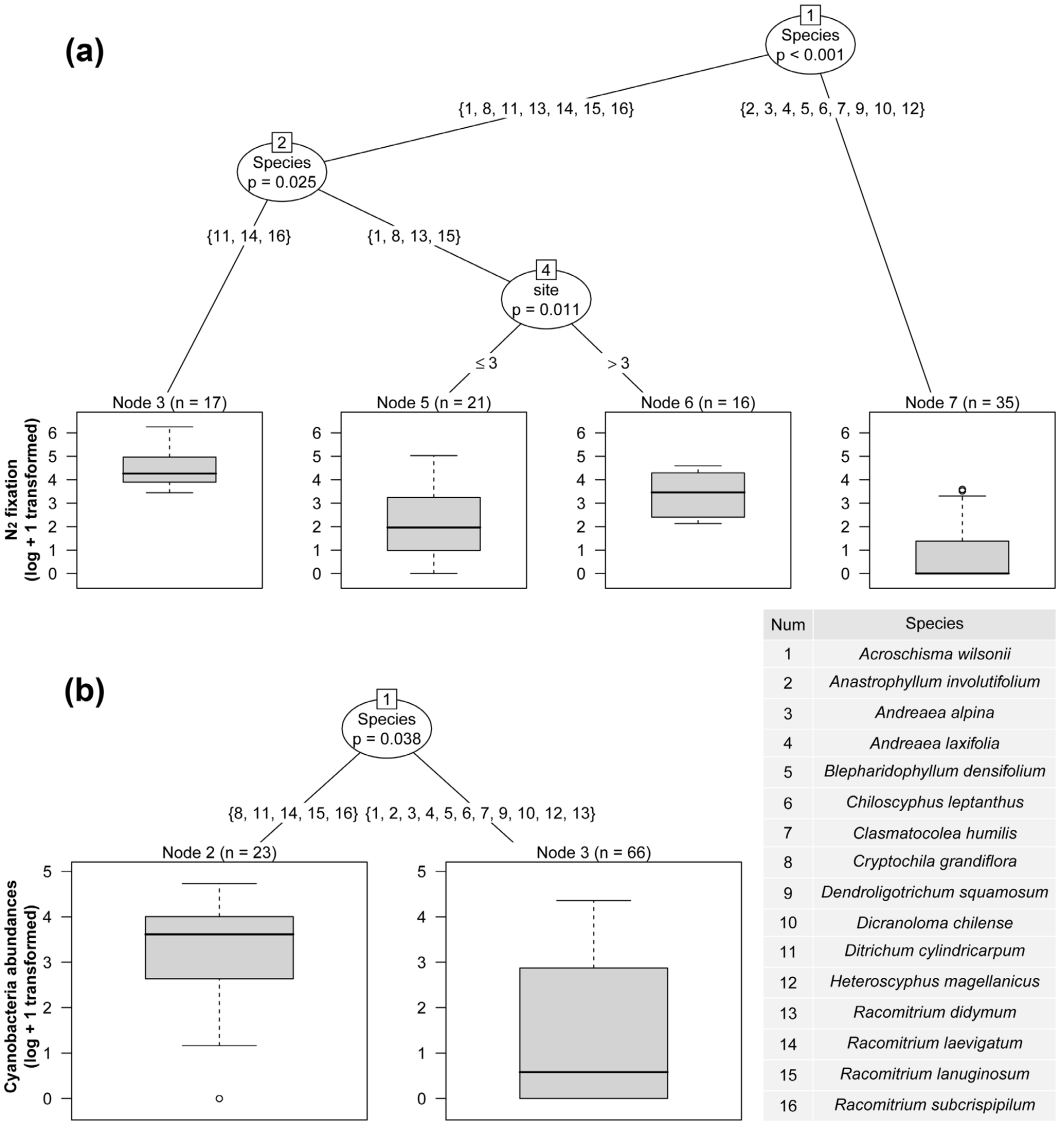
Conditional inference tree of: a) N_2_ fixation capacity (nmol C_2_H_2_ g^−1^ dm bryo. h^−1^) and b) cyanobacteria abundances (nmol echinenone/bryophyte cm^2^) on bryophytes from both glaciers. Bubbles (nodes) indicate the variable with the strongest effect on the response, best split value, and associated probability (Bonferroni-adjusted α = 0.05). Boxplots (terminal nodes) summarize the rate of N_2_ fixation (log+1 transformed) or cyanobacteria abundances (log+1 transformed) in cases classified into each terminal node (n). Predictor variables included: bryophyte species identity (species effect), glacier locations (glacier effect) and the stage of ecological succession (sampling site effect). The bryophyte species name and their corresponded number are shown in the table within the graph.

The conditional inference tree (Fig. 4a) revealed that both species identity and the stage of ecological succession (sampling site), were important variables in the N_2_ fixation rates at species level (ARA, nmol C_2_H_2_ g^−1^ dm bryo. h^−1^) Three species of mosses (*Ditrichum cylindricarpum*, *Racomitrium subcrispipilum* and *R. laevigatum*) showed the highest rates of N_2_ fixation (Table 1 and node 3 in Fig. 4a); Intermediate N_2_ fixation rates were found for a second group of species (node 4), namely *Racomitrium didymium, R. lanuginosum, Acroschisma wilsonii* and the liverwort *Cryptochila grandiflora*. An increment of N_2_ fixation rates was detected between younger sites (node 5: sites 1–3) and older sites (node 6: sites 4 and 5) for node 4. However, a more detailed analysis by individual species, revealed that there was an effect of the stage of ecological succession on N_2_ fixation rates only on the two most common species sampled in this study, *R. didymium* and *A. wilsonii* (statistical data not shown). The remainder of the species (node 7) had either low N_2_ fixation rates as detected in *Dicranoloma chilense*, *Andreaea laxifolia, Andreaea alpina* and *Anastrophyllum involutifolium*, or zero N_2_ fixation rates, as in the liverworts *Clasmatocolea humilis, Blepharidophyllum densifolium, Chiloscyphus leptanthus, Heteroscyphus magellanicus* and the endohydric moss *Dendroligotrichum squamosum* (Table 1 and Fig. 4a). With our dataset, no effect was detected for glacier location (north *sv*. south) at species level.

**Table 1:** Mean (*N* = 3) N_2_ fixation rates (nmol C_2_H_2_ g^−1^ dm bryo. h^−1^) cyanobacteria abundance (nmol echinenone/bryophyte cm^2^) and cyanobacteria genera associated with each individual bryophyte species along both glacier chronosequences.

			Mean reduction rate (nmol C_2_H_2_ g^−1^ dm bryo. h^−1^)		Cyanobacteria abundance (nmol echin. cm^−2^)	Cyanobacteria identification
Glacier	Site	Bryophyte species	Mean	Range		
SG	1	*Ditrichum cylindricarpum*	283.4	55–520	51.5 (3.2)	*N. commune*. *Leptolymbia sp*
SG	2	*Ditrichum cylindricarpum*	270.1	70–455	53.3 (8.2)	*N. commune*
SG	4	*Racomitrium subcrispipilum*	86.7	43–144	30.7 (12.1)	*N. commune*
SG	5	*Racomitrium subcrispipilum*	81.6	49–142	40.3 (36.2)	*N. commune.* Unidentified Nostocaceae *Stigonema*
NG	3	*Racomitrium laevigatum*	63.5	33–125	8.5 (6.9)	*N. commune*
NG	4	*Racomitrium laevigatum*	59.9	30–101	37.2 (9.4)	Unidentified Nostocaceae
NG	2	*Racomitrium didymum*	7.7	3–16	14.5 (14.5)	*N. commune*
NG	3	*Racomitrium didymum*	30.7	12–56	8.5 (5.3)	Unidentified Nostocaceae
NG	4	*Racomitrium didymum*	51.6	22–80	12.4 (7.1)	*Stigonema*
SG	3	*Racomitrium didymum*	0.0	0	0.3 (0.3)	*N. commune*
SG	4	*Racomitrium didymum*	20.7	7–34	21.5 (10.7)	Unidentified Nostocaceae
SG	5	*Racomitrium didymum*	66.6	53–75	39.4 (19.6)	*N. commune. Stigonema*
NG	1	*Andreaea laxifolia*	8.7	0–26	15.8 (15.5)	*Non found*
NG	3	*Andreaea laxifolia*	62.8	2–151	18.8 (9.6)	Unidentified Nostocaceae
NG	3	*Cryptochila grandiflora*	52.6	3–124	17.1 (4.5)	*N. commune*
NG	2	*Acroschisma wilsonii*	1.4	0–3	11.8 (6.0)	*Non found*
NG	3	*Acroschisma wilsonii*	6.1	1–14	0.0	Unidentified Nostocaceae
SG	3	*Acroschisma wilsonii*	25.7	25–40	12.5 (12.5)	*N. commune*
SG	4	*Acroschisma wilsonii*	18.4	9–28	19.5 (6.3)	Unidentified Nostocaceae
SG	5	*Acroschisma wilsonii*	39.8	8–72	9.9 (4.9)	Unidentified Nostocaceae
NG	4	*Racomitrium lanuginosum*	39.2	10–98	76.5 (22.0)	*Stigonema*
NG	3	*Dicranoloma chilense*	19.2	9–33	2.7 (2.7)	Unidentified Nostocaceae
NG	5	*Dicranoloma chilense*	2.9	0–9	5.9 (5.9)	*Non found*
NG	5	*Anastrophyllum involutifolium*	3.5	0–11	5.7 (5.1)	*Non found*
SG	4	*Clasmatocolea humilis*	1.5	0–3	15.2 (4.5)	*Stigonema*
NG	2	*Andreaea alpina*	0.9	0–3	2.7 (2.7)	*Non found*
NG	5	*Blepharidophyllum densifolium*	0.0	0	6.1 (6.1)	*Microcoleus*
SG	5	*Chiloscyphus leptanthus*	0.0	0	4.8 (4.8)	*Non found*
NG	1	*Dendroligotrichum squamosum*	0.0	0	3.2 (3.2)	*Non found*
NG	2	*Dendroligotrichum squamosum*	0.0	0	2.5 (2.5)	*Non found*
NG	5	*Heteroscyphus magellanicus*	0.0	0	0.1 (0.1)	*Non found*

In both glaciers the younger site (site 1) correspond to early succession site close to the glacier front whereas the older site (site 5) was settled within *Nothofagus* forests. Sites 3–4 represent intermediate stages of ecological succession. No species were sampled at site 0. SE for cyanobacteria abundances is shown in brackets. NG: north-side glacier; SG: south-side glacier of the mountain range.

**Figure 5:**
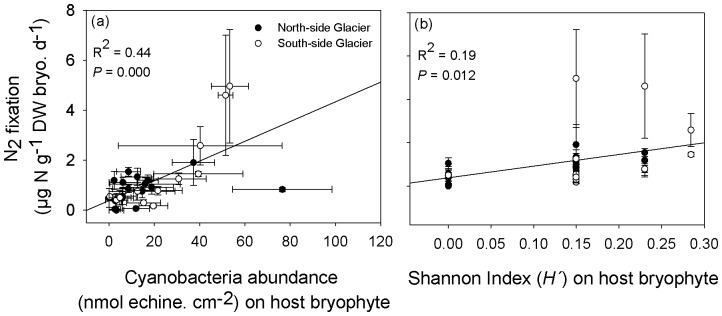
Relationship between N_2_ fixation and the abundance and diversity of epiphytic cyanobacteria. Relationship between N_2_ fixation (µg N g^−1^ DW bryo. d^−1^) on each individual bryophyte species and the abundance (nmol echine. cm-2) and diversity (Shannon Index) of their host cyanobacteria. Bryophyte species from both glaciers are included. The results of the linear regression analysis are shown in the upper left side of the figure.

The conditional inference tree (Fig. 4b) also showed a significant effect of bryophytes species identity on cyanobacteria relative abundance (nmol echinenone/cm^2^ bryophyte) with the highest colonization found in the mosses *Ditrichum cylindricarpum*, *Racomitrium subcrispipilum*, *R. laevigatum, R. lanuginosum* and the liverwort *Cryptochila grandiflora* (Table 1 and node 2 in Fig 4b). Variability of N_2_ fixation rates between bryophyte species was best explained by changes in epiphytic cyanobacteria abundance (positive linear regression: *R^2^* = 0.44, *P*<0.001, Fig. 5a) rather than the Shannon diversity index of cyanobacteria on host bryophytes (positive linear regression: *R^2^* = 0.19, *P* = 0.012, Fig. 5b).

### Community structure of epiphytic cyanobacteria

**Figure 6:**
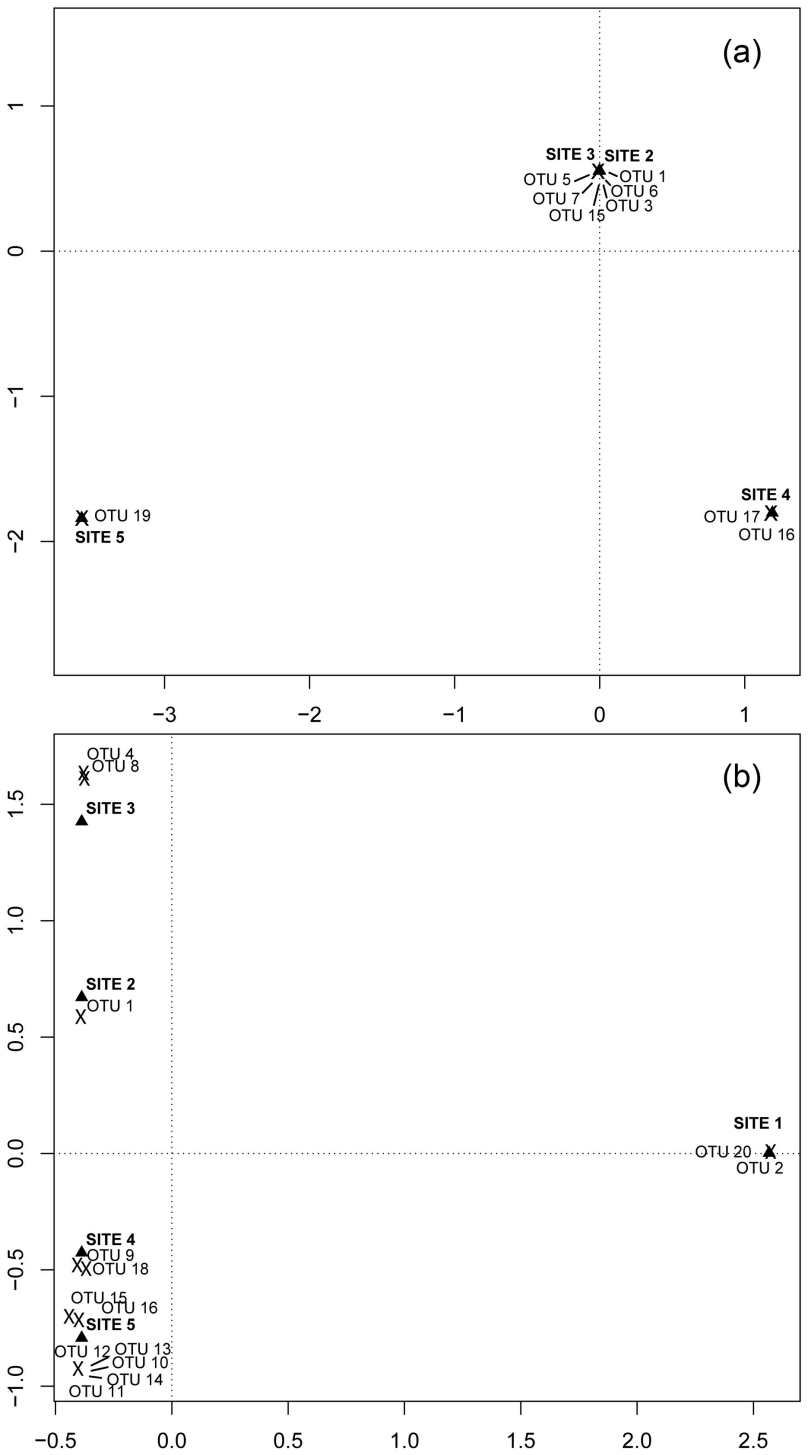
Relationships between cyanobacteria and the stages of ecological succession. Relationships between OTUs of epiphytic cyanobacteria obtained at a 0.01% cut-off and the different stages of ecological succession (triangles) -the younger site (site 1) correspond to bare ground close to the glacier front whereas the older site (site 5) correspond to *Nothofagus* forests- in the north-side glacier (a) and south-side glacier (b) after correspondence analysis. Site 1 is not included in (a) because cyanobacteria were not found epiphytically on bryophytes at that site. For OTUs identification please see Fig. 2.

The Shannon index of epiphytic cyanobacteria OTUs per site (*H′_site_*) at NG rose from 0.29 to a maximum of 0.96 at 26 years followed by a steady decline to original level at the oldest site. At SG, *H′_site_* increased sharply from 0.38 and 0.32 at the two youngest sites to 1.37 at the oldest site within *Nothofagus* forest (Fig. 3h). Correspondence analysis showed a clear relationship between cyanobacteria genera and the stage of ecological succession at both glaciers. At NG (Fig. 6a), sites 2 and 3 share similar cyanobacteria communities; *Nostoc commune* was present at site 2 (OTU 1 & 3) and site 3 (OTU 6) whereas unidentified Nostocaceae was confined to site 3 (OTU 5, 7 & 15) and site 4 (OTU 17); *Stigonema spp* was found just at site 4 (OTU 16) and *Microcoleus spp.* (OTU 19) at site 5. At SG (Fig. 6b), all sites differed in their cyanobacteria communities, *Nostoc commune* was present at sites 1 (OTU 2), site 2 (OTU 1), site 3 (OTU 4 & 8) and site 5 (OTU 11); unidentified Nostocaceae at site 4 (OTU 9) and site 5 (OTU 15 & 12) and *Stigonema spp.* at site 4 (OTU 18) and site 5 (OTU 16, 13, 10 & 14). *Leptolyngbya spp.* (OTU20) was found at site 1. No relationship was observed between the genus of cyanobacteria and bryophyte species (Table 1 and Fig. S2).

## Discussion

### Patterns of soil properties and N_2_ fixation related parameters along chronosequences

The trend observed in soil development followed the classic sequence reported for glacier foreland chronosequences [57], [58]. Soil pH fell and total C and N contents increased with surface age along both chronosequences. However, the time required differs significantly to rates reported in other proglacial areas. Soils developed from barren rocks to Histosol, an organic soil, with 39% C in SG and 35% C in NG after 34 and 80 years of soil exposure, respectively (Fig. 3b). This contrasts with 2% C reported in other studies reached 80 years after deglaciation [57]. Total soil N increased from very low or undetectable values at the youngest sites to 1.5% in SG and 0.99% in NG in the forest soil over this short period, again contrasting with concentration of 0.2% TN after 80 years exposure observed in the Central Alps [57]. These results suggest that conditions for vegetation succession are more favourable under the oceanic climatic conditions of Southern Hemisphere high latitude regions, such as Tierra del Fuego.

Soil development along the two chronosequences, from bare ground to full forest, showed similarities in soil pH and total C and N concentration, indicating similar stages of soil development at both sites (Fig. 3a,b,c and Fig. S1). However, the time-required for C and N accumulation was much less at SG (Fig. 3d). In addition, after 4 and 7 years of soil exposure extractable inorganic N concentration was already much higher in the soil at SG (13.42 mg kg^−1^ and 12.51 mg kg^−1^ respectably, Fig. 3d) than at NG, where values remained low (0.16 to 8.23 mg kg^−1^) even after 26 years of soil exposure. We also found the highest rates of N_2_ fixation and cyanobacteria abundance in the pioneer terricolous moss *Ditrichum cylindricarpum* at 4 and 7 years soil exposure in SG (Fig 3e,f Table 1), where it forms a dense carpet in the soil (Fig. 1b). By contrast, in NG biological N_2_ fixation and epiphytic cyanobacteria abundance on bryophytes was very low during the early stages of succession (Fig. 3e,f). It is likely that higher annual rainfall on SG is associated with higher rates of N_2_ fixation on bryophyte-cyanobacteria associations in young soils, since fixation of N is strongly controlled by moisture availability [30]. Earlier studies have shown the importance of free living colonies of diazotrophs for C and N inputs during early stages of succession in glacial forelands [59]–[62] which could contribute to nutrient accumulation at our sites. Fixation of N by cyanobacteria associated with bryophytes that colonize soil surfaces very early after glacier retreat, as shown by our results, could also help accelerate soil development in recently deglaciated areas under wetter conditions.

As succession proceeds in SG, terricolous mosses are replaced by perennial herbs like *Gunnera magellanica,* which forms symbiotic associations with the cyanobacterial genus Nostoc [63], and becomes the dominant species together with *Uncinia tenuis,* which are likely to be a major factor promoting C and N accumulation on soil. In intermediate-aged sites, mosses are confined to rock outcrops (Fig. 1b) where, together with lichen species like *Placopsis perrugosa* [64], form a thick layer of organic material on rock surfaces (Fig. 1b). Despite rock outcrops possibly being a less favourable surface for N_2_ fixation, we found high rates of fixation, abundance and diversity of epiphytic cyanobacteria on species like *Racomitrium subcrispipilum, Racomitrium didymium* and *Acroschisma wilsonii* which formed moss mats on rock surfaces at intermediate-aged sites (19 years old sites in SG) and even within *Nothofagus* forest (Table 1 and Fig. 3h). However, at the drier side of the mountain range where NG flows, intermediate-aged sites are dominated by the dwarf shrub *Empetrum rubrum* which allows the development of dense mats of terricolous bryophyte species such as *Racomitrium lanuginosum, R. laevigatum* and *Cryptochila grandiflora* (Fig. 1b). It is at this stage of succession that we found the highest rate of N_2_ fixation and cyanobacteria colonization on bryophytes at NG (Fig. 3e,f and Table 1). In this drier area dominated by dwarf shrub vegetation, cyanobacteria in association with bryophytes are possibly one of the main sources of fixed N, since cyanobacteria in the soil decrease with increasing vegetation development, and the occurrence of N-fixing Nostoc host, *Gunnera magellanica*, is reduced under drier conditions.

It remains unclear, however, how the newly fixed N in the bryophyte-cyanobacteria system becomes available to vascular plants. We consider two pathways, described by [30], which may occur during primary succession in our study area. First, direct transfer of N from the moss system to vascular plants by mycorrhizal linkages: most vascular plant species in the study area form mycorrhizal association (e.g. ectomycorrhizal association with *Nothofagus* species or ericoid mycorrhizal with *Empetrum* spp.). Second, release of the newly fixed N retained in the bryophyte-cyanobacteria system after disturbance events, for example after membrane damage from desiccation following wet/dry cycles [65], [66].

Overall, glacier forelands in Tierra del Fuego show fast rates of soil transformation, implying large quantities of N inputs to allow fast vegetation succession. Our results highlight that bryophyte-cyanobacteria associations have the potential to contribute to N accumulation and cycling in these areas with very low inputs of N from atmospheric deposition.

### Differences on bryophyte N_2_ fixation capacity and cyanobacteria abundance

Few studies have investigated interspecific or intraspecific variation in N_2_ fixation capacity on bryophytes, particularly in the context of post-glacial primary succession. In our study, most bryophyte species have the ability to support an epiphytic flora of cyanobacteria. However, N_2_ fixation rates varied between and within bryophyte species (Table 1). In line with results reported in previous studies [5], we detected differences in N_2_ fixation rates between mosses and liverworts. Five of the six studied liverworts showed no or very low rate of N_2_ fixation and cyanobacteria abundance (Table 1), and only one species, the terricolous liverwort *Cryptochila grandiflora,* showed high fixation rates (52.6 nmol C_2_H_2_ g^−1^ dm bryo. h^−1^) and cyanobacteria colonization. Among moss species, *Dendroligotrichum squamosum* also showed no N_2_ fixation or cyanobacteria colonization, possibly due to their rigid, water repellent leaves which may confer less favorable moisture conditions. However, morphology or habitat could not explain variations found among other moss species. Both terricolous (*Ditrichum cylindricarpum*, *Racomitrium laevigatum*, *R. lanuginosum*) and saxicolous species (*Racomitrium subcrispipilum, R. didymum, Acroschisma wilsonii*) showed high N_2_ fixation rates (over 40 nmol C_2_H_2_ g^−1^ dm bryo. h^−1^) compared to rates reported in Arctic [8] and sub-Antarctic [16] ecosystems. Regarding intraspecific variations, we observed that the rates of N_2_ fixation of the saxicolous mosses *Racomitrium didymium* and *Acroschisma wilsonii* (the most frequently sampled species in the study area) were affected by the stages of ecological succession. The rate of N_2_ fixation significantly increases on *R. didymium* and *Acroschisma wilsonii* populations that grow at sites that have been longer deglaciated, reflecting growth of the cyanobacterial population as vegetation develops when growing epiphytically on these mosses.

### Community structure of epiphytic cyanobacteria

In the present study, we also aimed to evaluate the influence of cyanobacteria community structure on bryophyte N_2_ fixation rates. We found that abundance of epiphytic cyanobacteria appears to be a better factor explaining differences in N_2_ fixation rates (Fig. 5) than epiphytic cyanobacteria diversity on host bryophyte species (Fig. 5). It is also noteworthy that *Nostoc commune*-associated bryophyte species, both mosses and liverworts, showed the highest rate of N_2_ fixation (Table 1), which is in agreement with the suggestion that *Nostoc commune,* in particular, is an important contributor of soil N in both polar regions [32].

Specific affinities of individual cyanobacteria genera with particular bryophyte species were not apparent, as the same bryophyte species can support the three main cyanobacteria genera found here (*Nostoc*, unidentified Nostocaceae group and *Stigonema*) (Table 1 and Fig. S2). However, a relationship between cyanobacteria genera and the different stages of succession was apparent in both glaciers. With increasing age along the chronosequences, cyanobacteria shifted from: *Nostoc commune*-dominated communities at young sites; to unidentified Nostocaceae-dominated communities at intermediate sites; and finally to *Stigonema* spp-dominated communities at older sites (Fig. 6). Such procession correlated with the soil development trends found here and contrasts with results observed in mature boreal forests where moss species identity serves as an important determinant of cyanobacterial communities that inhabit mosses [22]. With our data set, our results suggest that the stage of succession acts as a strong driver in determining the composition of epiphytic cyanobacteria on bryophytes during primary succession. Despite this similarity between the two glaciers, diversity of epiphytic cyanobacteria at SG was higher than at NG and increased with time since deglaciation (Fig. 3h), reflecting more favourable conditions for cyanobacteria growth on the wetter southern side of Cordillera Darwin.

### Conclusions

We have compared, from a multidisciplinary perspective, chronosequences in front of two receding glaciers with contrasting climatic conditions (wetter *vs* drier) and differing rates of ecological succession in Tierra del Fuego (Chile). In doing so, we have shown that bryophyte-cyanobacteria associations have the potential to contribute to N accumulation very early after glacier retreat, a process that could help promote soil development. At intermediate-aged sites, and under drier conditions, bryophytes can grow among dwarf shrub plants, forming dense mats that host cyanobacteria species and exhibit high N_2_ fixation rates, contributing to N accumulation and cycling. We also found that differences in N_2_ fixation capacity between bryophyte species were primarily driven by the abundance of epiphytic cyanobacteria rather than cyanobacterial community composition. Most liverworts showed low colonization, whilst in mosses rates of N_2_ fixation did not exhibit consistent differences across life forms and habitat (saxicolous *vs* terricolous). Finally, we identified that the main factor structuring community composition of epiphytic cyanobacteria was the stage of ecological succession, with no relationship to host species identity.

## Supporting Information

Figure S1 **Scores variation of the general factor component of soil development along the glacier chronosequence in the north-side (black bars) and south-side (white bars) of Cordillera Darwin.** Sites with higher loadings reflect a more developed soil. The general factor component of soil development was obtained by reduction of soil pH and total soil carbon and nitrogen by principal component analysis. Mean values (± SE) of three replicate transect are shown.
(DOC)

Figure S2 **Correspondence analysis between OTUs of cyanobacteria obtained at a 0.01% cut-off and each individual bryophyte species, just the six most abundance bryophyte species with cyanbacteria colonies were used in this analysis, identification of OUTs can be see in** **Fig.2** **.**
(DOC)

Table S1 **Changes in bryophyte species along two proglacial chronosequence at the Cordillera Darwin (Tierra del Fuego, Chile).**
(DOC)

Table S2 **Gen Bank accession numbers corresponding to species and specimens used for tree inference.**
(DOC)

Appendix S1 **Calculation of different vial headspaces for ARA and ^15^N_2_ uptake essays.**
(DOC)
