# Correction: Bryophyte-Cyanobacteria Associations during Primary Succession in Recently Deglaciated Areas of Tierra del Fuego (Chile)

**DOI:** 10.1371/journal.pone.0108759

**Published:** 2014-09-18

**Authors:** The PLOS ONE Staff

The following information is missing from the Funding section: MA-C benefited from a postdoctoral Juan de la Cierva grant of the Ministerio de Ciencia e Innovación of Spain.

There are errors in the Author Contributions. The correct contributions are: Performed the experiments: MA-C SPO ADLR MC MTDLC ROH. Analyzed the data: MA-C SPO NT. Carried out sampling field work: AP SPO TGAG. Selected field sites: DP RR LGS.
