# Supplementary material for: Bryophyte-Cyanobacteria Associations during Primary Succession in Recently Deglaciated Areas of Tierra del Fuego (Chile)
Source: PLoS One. 2014 May 12;9(5):e96081. doi: 10.1371/journal.pone.0096081 (PMC4018330; doi:10.1371/journal.pone.0096081)
Supplement: Figure S1 — Scores variation of the general factor component of soil development along the glacier chronosequence in the north-side (black bars) and south-side (white bars) of Cordillera Darwin. Sites with higher loadings reflect a more developed soil. The general factor component of soil development was obtained by reduction of soil pH and total soil carbon and nitrogen by principal component analysis. Mean values (± SE) of three replicate transect are shown. (DOC) [file pone.0096081.s001.doc]

**Supporting Information, Figure S1**

Glacier exposure: *NS*

Sampling site: *P* < 0.001

Glacier x site : *NS*

0

1

2

3

4

5

**Fig. S1** Scores variation of the general factor component of soil development along the glacier chronosequence in the north-side (black bars) and south-side (white bars) of Cordillera Darwin. Sites with higher loadings reflect a more developed soil. The general factor component of soil development was obtained by reduction of soil pH and total soil carbon and nitrogen by principal component analysis. Mean values (± SE) of three replicate transect are shown.
