# Supplementary material for: Bryophyte-Cyanobacteria Associations during Primary Succession in Recently Deglaciated Areas of Tierra del Fuego (Chile)
Source: PLoS One. 2014 May 12;9(5):e96081. doi: 10.1371/journal.pone.0096081 (PMC4018330; doi:10.1371/journal.pone.0096081)
Supplement: Figure S2 — Correspondence analysis between OTUs of cyanobacteria obtained at a 0.01% cut-off and each individual bryophyte species, just the six most abundance bryophyte species with cyanbacteria colonies were used in this analysis, identification of OUTs can be see in Fig.2 . (DOC) [file pone.0096081.s002.doc]

**Supporting Information, Figure S2**


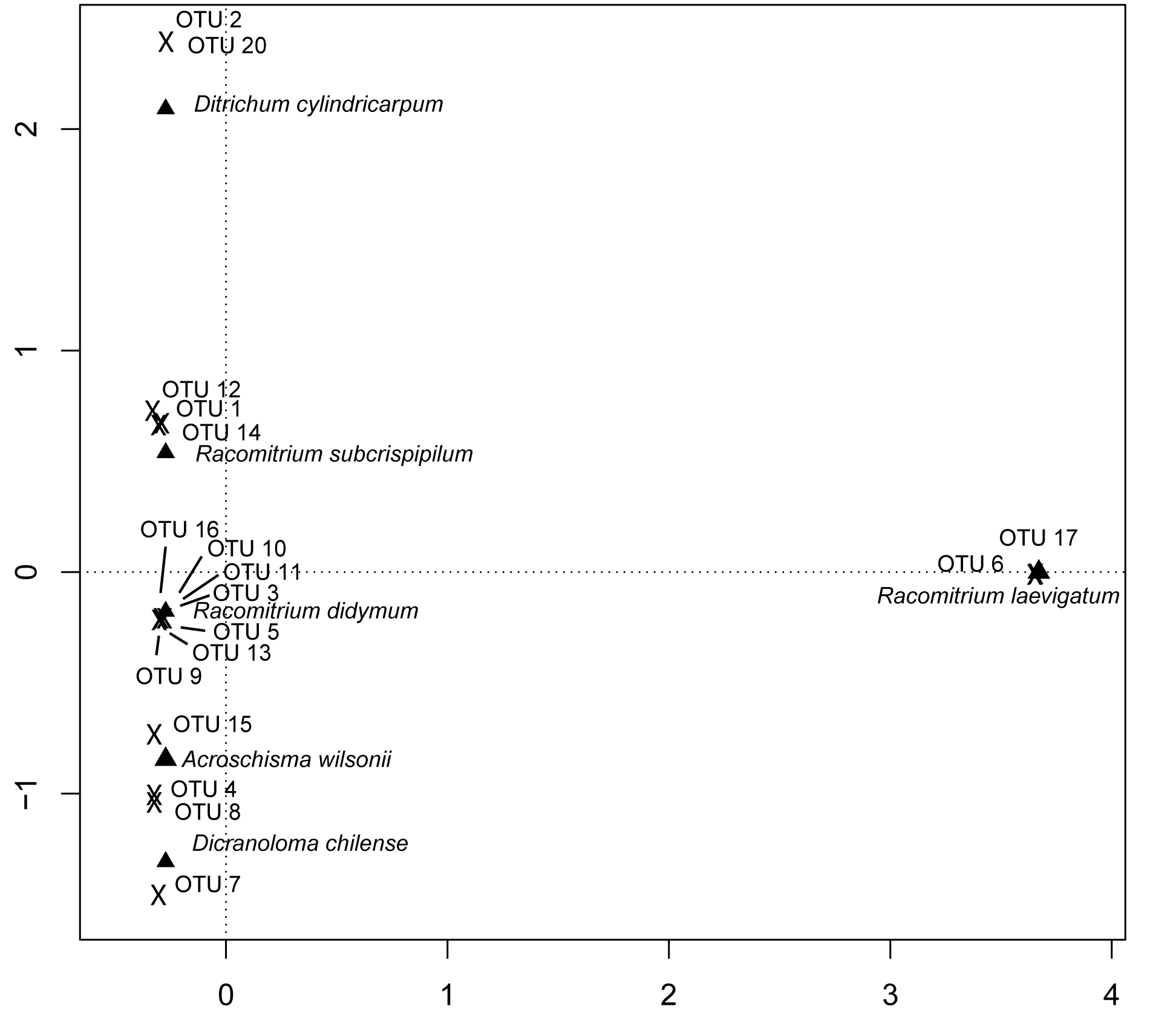


**Fig S2.** Correspondence analysis between OTUs of cyanobacteria obtained at a 0.01% cut-off and each individual bryophyte species, just the six most abundance bryophyte species with cyanbacteria colonies were used in this analysis, identification of OUTs can be see in Fig.2
