# Supplementary material for: Bryophyte-Cyanobacteria Associations during Primary Succession in Recently Deglaciated Areas of Tierra del Fuego (Chile)
Source: PLoS One. 2014 May 12;9(5):e96081. doi: 10.1371/journal.pone.0096081 (PMC4018330; doi:10.1371/journal.pone.0096081)
Supplement: Table S1 — Changes in bryophyte species along two proglacial chronosequence at the Cordillera Darwin (Tierra del Fuego, Chile). (DOC) [file pone.0096081.s003.doc]

**Supporting Information, Table S1**

**Table S1**. Changes in bryophyte species along two proglacial chronosequence at the Cordillera Darwin (Tierra del Fuego, Chile).

|  | Soil (surface) age | | | | | |
| --- | --- | --- | --- | --- | --- | --- |
| **North-side glacier** | 5 years | 18 years | 20 years | 26 years | 66 years | 80 years |
| *Dendroligotrichum squamosum* | *-* | X | X |  |  |  |
| *Andreaea laxifolia* | *-* | X |  | x |  |  |
| *Acroschisma wilsonii* | *-* |  | X | x |  |  |
| *Andreaea alpina* | *-* |  | X |  |  |  |
| *Racomitrium didymum* | *-* |  | *x* | x | *x* |  |
| *Racomitrium laevigatum* | *-* |  |  | *x* | x |  |
| *Cryptochila grandiflora* | *-* |  |  | *x* |  |  |
| *Dicranoloma chilense* | *-* |  |  | x |  | X |
| *Racomitrium lanuginosum* | *-* |  |  |  | *x* |  |
| *Heteroscyphus magellanicus* | *-* |  |  |  |  | X |
| *Blepharidophyllum densifolium* | *-* |  |  |  |  | *x* |
| *Anastrophyllum involutifolium* | *-* |  |  |  |  | X |
| **South-side glacier** | 1 years | 4 years | 7 years | 10 years | 19 years | 34 years |
| *Ditrichum cylindricarpum* | *-* | *x* | *x* |  |  |  |
| *Acroschisma wilsonii* | *-* |  |  | *x* | *x* | x |
| *Racomitrium didymum* | *-* |  |  | *x* | x | *x* |
| *Racomitrium subcrispipilum* | *-* |  |  |  | *x* | *x* |
| *Clasmatocolea humilis* | *-* |  |  |  | *x* |  |
| *Chiloscyphus leptanthus* | *-* |  |  |  |  | X |
