# Supplementary material for: Bryophyte-Cyanobacteria Associations during Primary Succession in Recently Deglaciated Areas of Tierra del Fuego (Chile)
Source: PLoS One. 2014 May 12;9(5):e96081. doi: 10.1371/journal.pone.0096081 (PMC4018330; doi:10.1371/journal.pone.0096081)
Supplement: Table S2 — Gen Bank accession numbers corresponding to species and specimens used for tree inference. (DOC) [file pone.0096081.s004.doc]

**Supporting Information, Table S2**

| **Taxon** | **nuSSU Accession Number** |
| --- | --- |
| ***Anabaena oscillarioides*** | AJ630426 |
| ***Anabaena* sp.** | EF568904 |
| ***Anabaena* sp.** | EF583861 |
| ***Anabaena* sp.** | FJ982323 |
| ***Leptolyngbya* *frigida*** | AY493612 |
| ***Leptolyngbya frigida*** | AY493610 |
| ***Leptolyngbya tenuis*** | GQ859652 |
| ***Microcoleus* sp.** | EF654061 |
| ***Microcoleus* sp.** | EU586738 |
| ***Microcoleus* sp.** | EU586739 |
| ***Nostoc commune*** | EU178142 |
| ***Nostoc commune*** | AB088375 |
| ***Nostoc commune*** | AY577536 |
| ***Nostoc commune*** | AB113665 |
| ***Nostoc edaphicum*** | HQ700837 |
| ***Nostoc punctiforme*** | AF027655 |
| ***Nostoc punctiforme*** | GQ287652 |
| ***Nostoc* sp** | AM711539 |
| ***Nostoc* sp** | AM711531 |
| ***Nostoc* sp.** | GU254536 |
| ***Nostoc* sp.** | GU062469 |
| ***Nostoc* sp.** | AB088405 |
| ***Nostoc* sp.** | AF506248 |
| ***Nostoc* sp.** | AY328896 |
| ***Phormidium* sp.** | AM398795 |
| ***Scytonema* cf. *chiastum*** | JN565280 |
| ***Stigonema* *ocellatum*** | GQ354275 |
| ***Stigonema* *ocellatum*** | AJ544082 |
| ***Trichodesmium* *erythraeum*** | AF013030 |

**Table S2**. Gen Bank accession numbers corresponding to species and specimens used for tree inference.
