# Supplementary material for: Bryophyte-Cyanobacteria Associations during Primary Succession in Recently Deglaciated Areas of Tierra del Fuego (Chile)
Source: PLoS One. 2014 May 12;9(5):e96081. doi: 10.1371/journal.pone.0096081 (PMC4018330; doi:10.1371/journal.pone.0096081)
Supplement: Appendix S1 — Calculation of different vial headspaces for ARA and 15N2 uptake essays. (DOC) [file pone.0096081.s005.doc]

**Supporting Information, Appendix S1**

*Calculation of different vial headspaces for ARA and ^15^N_2_ uptake essays*

Enrichment was determined individually for each sample bearing in mind the different vial headspaces. Calculation of the headspaces was performed base on the volume occupied by each sample according to the dry weigh used per sample. Bryophyte shoots (~2-4 mg of dry tissue per sample depending on the species) was used to calculate the ration between cm^3^ of bryophyte shoot / mg dry mass in each individual species. Volume of fresh bryophyte shoots was calculated according to their different morphologies, mosses leaves are usually packed and spirally arranged along the stem forming a cylinder-like shape whereas most liverworts have dorsiventral morphology forming a rectangle-like shape. We estimate moss shoot volume by the cylinder volume formula (*V* = *πxr^2^xh*) and liverwort shoot volume by the rectangle volume formula (*V* = *lxwxh*).
